# SRCT: Structure-Preserving Method for Sub-Meter Remote Sensing Image Super-Resolution

**DOI:** 10.3390/s26020733

**Published:** 2026-01-22

**Authors:** Tianxiong Gao, Shuyan Zhang, Wutao Yao, Erping Shang, Jin Yang, Yong Ma, Yan Ma

**Affiliations:** 1 Aerospace Information Research Institute, Chinese Academy of Sciences, Beijing 100094, China; gaotianxiong23@mails.ucas.edu.cn (T.G.); yaowt@aircas.ac.cn (W.Y.); shangep@aircas.ac.cn (E.S.); yangjin@aircas.ac.cn (J.Y.); mayan@aircas.ac.cn (Y.M.); 2 School of Electronic, Electrical and Communication Engineering, University of Chinese Academy of Sciences, Beijing 100049, China; 3 State Key Laboratory of Media Convergence Production Technology and Systems, Beijing 100803, China

**Keywords:** remote sensing, super-resolution reconstruction, structure preservation, deep learning

## Abstract

To address the scarcity of sub-meter remote sensing samples and structural inconsistencies such as edge blur and contour distortion in super-resolution reconstruction, this paper proposes SRCT, a super-resolution method tailored for sub-meter remote sensing imagery. The method consists of two parts: external structure guidance and internal structure optimization. External structure guidance is jointly realized by the structure encoder (SE) and structure guidance module (SGM): the SE extracts key structural features from high-resolution images, and the SGM injects these structural features into the super-resolution network layer by layer, achieving structural transfer from external priors to the reconstruction network. Internal structure optimization is handled by the backbone network SGCT, which introduces a dual-branch residual dense group (DBRDG): one branch uses window-based multi-head self-attention to model global geometric structures, and the other branch uses lightweight convolutions to model local texture features, enabling the network to adaptively balance structure and texture reconstruction internally. Experimental results show that SRCT clearly outperforms existing methods on structure-related metrics, with DISTS reduced by 8.7% and LPIPS reduced by 7.2%, and significantly improves reconstruction quality in structure-sensitive regions such as building contours and road continuity, providing a new technical route for sub-meter remote sensing image super-resolution reconstruction.

## 1. Introduction

The rapid development of remote sensing technology has enabled the acquisition of large amounts of remote sensing data. High-resolution remote sensing imagery is widely used in construction planning [1], disaster assessment [2], agricultural production [3], and other fields [4,5]. Its precise texture information and rich ground object information provide a reliable data foundation for fine-grained analysis and decision-making. In particular, sub-meter remote sensing imagery can be widely applied in urban refined management, precision agriculture, and environmental monitoring. However, high-resolution imagery, especially sub-meter imagery, remains in short supply. This is mainly due to sensor limitations, imaging constraints, and the high cost of deploying dense satellite constellations. Super-resolution (SR) reconstruction technology reconstructs low-resolution (LR) images into high-resolution (HR) images through algorithms, thereby obtaining more precise ground object information and improving the accuracy of remote sensing applications. It is the most cost-effective approach to address the shortage of high-resolution data.

**Figure 1:**
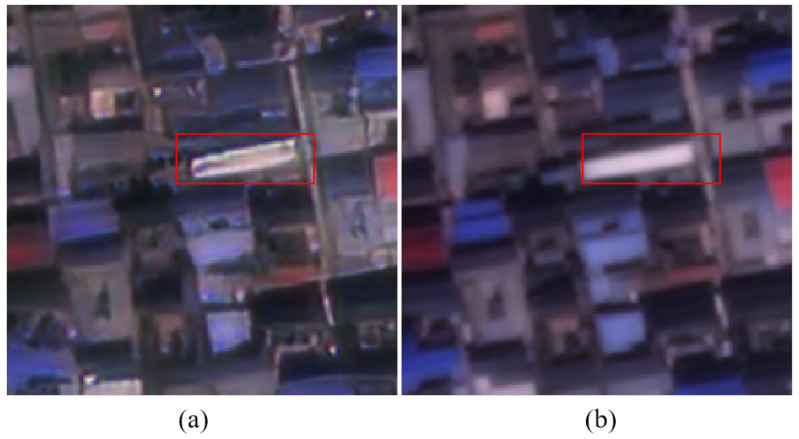
Comparison example between super-resolution results and HR in real remote sensing scenes: (**a**) reconstruction results of current mainstream super-resolution methods; (**b**) corresponding HR. The red box marks a representative building.

In super-resolution reconstruction, especially for large upscaling factors such as 4×, researchers face a fundamental challenge: how to preserve the integrity of complex ground object structures while recovering rich texture details. Remote sensing imagery covers various ground objects such as urban areas, farmland, and mountains, with significantly different texture characteristics and multi-scale structural features. As shown in Figure 1, urban buildings typically exhibit regular geometric structures, and super-resolution models must accurately restore these structures while avoiding aliasing, blurring, or deformation. In the HR image in (b), the target building has a clear and regular rectangular contour, whereas the reconstruction result of current mainstream super-resolution methods in (a) shows jagged edges and shape distortion, failing to preserve the true geometric structure of the building. In contrast, natural landscapes such as farmland and mountains have irregular textures and may contain many repetitive patterns (e.g., crops) or complex terrain undulations, where the model needs to capture fine details without distorting shapes. This imposes stricter requirements on super-resolution reconstruction models: when confronted with real complex scenes, they must simultaneously recover fine texture details and maintain the structural integrity of ground objects. To achieve high-precision super-resolved images, current research efforts mainly focus on super-resolution models based on convolutional neural networks (CNNs), generative adversarial networks (GANs), and Transformers.

CNN-based super-resolution methods began with SRCNN proposed by Dong et al. [6], which first applied CNNs to learn the mapping from low-resolution to high-resolution images, outperforming traditional methods. Subsequent improvements include the following: FSRCNN [7] introduced transposed convolution to reduce computational cost; VDSR [8] adopted residual learning and gradient clipping to alleviate gradient problems; DRRN [9] and DRCN [10] deepened networks through residual connections; EDSR [11] removed BN layers and introduced residual scaling to improve training stability; RCAN [12] introduced channel attention to enhance channel discrimination; DRN [13] proposed a dual-regression strategy to constrain the solution space; LTE [14] used implicit functions to reconstruct details in a continuous manner; and SMSR [15] achieved acceleration by learning sparse masks. These methods achieved some success in texture recovery, but they are limited by the local receptive field of CNNs, making it difficult to capture long-range dependencies in remote sensing images. They perform poorly when handling large-scale structures, often resulting in structural distortions such as edge blurring and straight-line distortion. Subsequent improvements, such as the dual-path structure of HAUNet [16], the residual split attention group by Chen et al. [17], and the edge extraction module by Wang et al. [18], have made progress, but the inherent locality limitations of CNNs have not been fully overcome.

To overcome the locality limitations of CNNs, researchers have introduced GAN-based methods. GANs were proposed by Goodfellow et al. [19] in 2014, initially for image generation, and later applied to various vision tasks. SRGAN [20] first introduced GANs into super-resolution, generating richer and clearer textures and details through adversarial training, making reconstruction results more visually realistic. Subsequent improvements include the following: Park et al. [21] introduced feature-domain discriminators to reduce high-frequency noise; ESRGAN [22] added dense connections and residual connections to the generator and used conditional discriminators to improve visual realism; Zhang et al. [23] constructed loss functions based on image quality ranking; and Ma et al. [24] introduced geometric structures of high-resolution images into generative adversarial networks. For remote sensing image super-resolution, CDGAN [25] proposed dual-input discriminators and added pairwise loss functions to address the discrimination difficulty caused by remote sensing images having far more low-frequency information than natural images; Ma et al. [26] constructed generators based on deep residual networks using a Wasserstein GAN with gradient penalty; Jia et al. [27] constructed MA-GAN, introducing pyramid multi-scale convolution, residual connections, and various attention mechanisms in the generator network; Dong et al. [28] proposed high-resolution reference image-assisted methods, mining high-frequency features of reference images through gradient-assisted feature alignment operations; SPSR [24] introduced gradient branches to provide structural priors; and WGSR [29] enhanced texture sensitivity through wavelet transforms. In addition, Tu et al. [30] fused Swin Transformer with CNN hybrid architectures, Jia et al. [27] integrated multiple attention mechanisms, and Wang et al. [31] designed multi-scale sliding window attention mechanisms, all effectively improving the reconstruction quality of remote sensing images. However, GAN frameworks also introduce new problems: unstable training, the tendency to generate false textures, and limitations in preserving key structures such as building contours and road networks in remote sensing images. Although the above improved methods enhance structure preservation through gradient branches, wavelet transforms, or attention mechanisms, they still face challenges when handling complex structures in remote sensing images.

In recent years, Transformer architectures have been introduced into super-resolution due to their strong global modeling capability. Transformer-based methods such as SwinIR [32] and IPT [33] capture global dependencies through self-attention mechanisms, effectively addressing the limitations of traditional CNN methods in large-scale scene reconstruction and showing clear advantages in complex texture regions, improving structure preservation to some extent. However, these methods also have inherent drawbacks: when models tend to amplify high-frequency details, high-frequency structural boundaries are easily weakened, leading to reduced accuracy of key geometric structures in remote sensing images. Additionally, deep networks still face information bottleneck effects, causing structural information to gradually degrade during transmission. To address these problems, subsequent researchers have proposed various improvement strategies. CATANet [34] introduces a content-aware token aggregation module to enhance perception of key structures and textures while maintaining global modeling capability. ACT-SR [35] proposes an aggregated connection Transformer, effectively enhancing the feature representation of remote sensing images and reducing artifacts by aggregating multi-level information from local to global, from original images to feature maps, and from spatial to channel. Xiao et al. [36] address the problem of redundant token representations in remote sensing scenes by dynamically selecting the top-k key tokens with the highest scores for each query, capturing the most critical information and reducing interference from irrelevant tokens. To expand the perspective of feature extraction, Shi et al. [37] fuse phase and amplitude features from the frequency domain into the image domain based on discrete Fourier transform (DFT) operators, providing a new approach for remote sensing image super-resolution reconstruction.

Beyond Transformer-based models, several approaches improve structure preservation in remote sensing super-resolution from different modeling paradigms. FreMamba [38] introduces the visual state space model Mamba into remote sensing super-resolution, leveraging its linear complexity to efficiently model long-range dependencies in large-scale imagery and reduce the computational burden associated with self-attention. Another related line explores dual-branch designs that couple complementary information to enhance reconstruction. CRefDiff [39] adopts a local–global dual-branch fusion strategy for reference-guided remote sensing super-resolution, while DBFNet [40] employs complementary branches within a residual dense framework and fuses them adaptively to improve restoration quality.

Across the above super-resolution methods, a common goal is to enhance perceptual quality and recover high-frequency details. CNN-based models excel at local texture reconstruction but struggle with long-range structure modeling. GAN-based methods improve visual sharpness yet may introduce hallucinated details and reduce geometric fidelity. Transformer-based approaches better capture long-range dependencies, but information can still attenuate during deep propagation. As a result, existing methods often fail to simultaneously restore fine textures and preserve the structural integrity of complex ground objects, largely because structure is not treated as an explicit signal with a dedicated shallow-to-deep transmission mechanism.

We build upon the ability of Transformer-based super-resolution methods to model long-range dependencies, and we are motivated to alleviate the information bottleneck that may arise during deep feature propagation in such architectures. In addition, dual-branch designs have demonstrated the effectiveness of coupling complementary information sources. Inspired by this principle, SRCT explicitly extracts structural cues and injects them hierarchically into the reconstruction network, establishing a dedicated pathway for structure transmission and mitigating structural attenuation in deep models.

Based on the above insights, we propose SRCT, a structure-preserving super-resolution method for remote sensing images. The method consists of three core components working together: a structure encoder (SE), a structure guidance module (SGM), and a structure-guided residual cross-Transformer (SGCT). Specifically, the SE extracts and preserves key structural information from the original image. The SGM delivers structural features to all levels of the super-resolution network through a two-stage injection strategy. The SGCT serves as the backbone network and employs a dual-branch residual dense group (DBRDG) tailored for remote sensing characteristics, effectively balancing the reconstruction needs of regular geometric structures and irregular textures. The entire framework maintains structural consistency while recovering texture details, providing a new technical approach for remote sensing image super-resolution reconstruction. The main contributions of this study are as follows:

(1) Structure Encoder (SE): A multi-level cascaded convolutional and spatial attention module is designed to effectively extract and preserve key structural information from the original image, while capturing both macro structural features and local texture patterns.
(2) Structure Guidance Module (SGM): A two-stage injection strategy is implemented, including feature initialization guidance and deep feature fusion. Through attention-enhanced hierarchical transmission of structural features, structural information is precisely injected into all levels of the super-resolution network, effectively alleviating the information bottleneck effect in Transformer models and ensuring the integrity of complex ground object structures.
(3) Dual-Branch Residual Dense Group (DBRDG) for Remote Sensing Characteristics: A dual-branch structure is innovatively designed with window-based multi-head self-attention in the main path and lightweight convolution in the residual path. This effectively balances the reconstruction needs of regular geometric structures and irregular textures in remote sensing images, achieving collaborative optimization of global structure modeling and local texture preservation. It is particularly suitable for handling the complex and diverse ground object types in remote sensing images.

## 2. Methods

### 2.1. Overview

**Figure 2:**
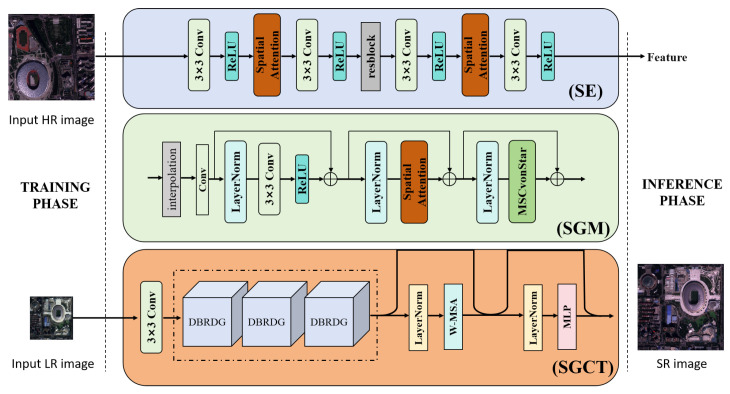
Structure-preserving remote sensing image super-resolution framework. The method consists of three core components, a structure encoder (SE), a structure guidance module (SGM), and a structure-guided residual cross-Transformer (SGCT), and includes both training and inference stages. The two dashed lines separate the inputs and outputs from the model modules, and the dashed box indicates that the Residual Dense Groups (DBRDGs) are composed of three cascaded DBRDG blocks.

As shown in Figure 2, the proposed structure-preserving super-resolution method for remote sensing images consists of three core components: a structure encoder (SE), a structure guidance module (SGM), and a structure-guided residual cross-Transformer (SGCT). The entire framework operates in two stages: training and inference. In the training stage, the left part of the framework shows the training dataset composed of HR–LR image pairs. The middle part illustrates the core processing flow of the model: high-resolution images first pass through the SE to extract structural features, and the SGM then transfers these features to the SGCT model for training. This design ensures that structural information can effectively guide the super-resolution reconstruction process. In the inference stage (right part of the framework), the LR input is processed by the trained SRCT model to generate an SR image that preserves structural integrity. By integrating structural guidance information, the SRCT model can recover texture details while accurately maintaining complex ground object structures in remote sensing imagery. This architecture effectively overcomes the limitations of traditional methods in structure preservation and is particularly suitable for diverse ground object types and complex scene structures in remote sensing images. With the SE extracting structural information, SGM providing structure guidance, and SRCT performing super-resolution reconstruction, the three components work together to balance texture detail restoration and structural integrity preservation.

### 2.2. Structure Encoder (SE)

**Figure 3:**
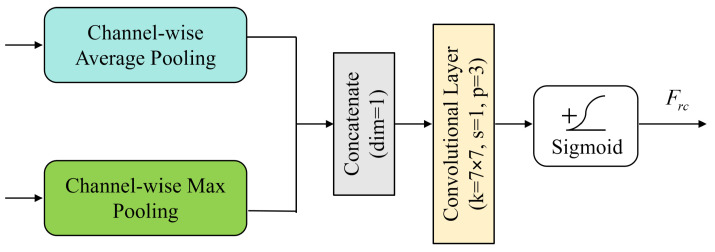
Structure of the spatial attention module. Channel-wise average pooling and max pooling are applied in parallel, concatenated along the channel dimension, and passed through a 7×7 convolution followed by a Sigmoid function to generate the spatial attention map $F_{rc}$ for emphasizing edge- and contour-dominant regions.

The structure encoder (SE) is used to extract and preserve key structural information from the original image. Different from designs where structural cues are expected to emerge implicitly from backbone features, the SE explicitly highlights edge- and contour-related responses and provides structure-aware features for hierarchical guidance, which helps mitigate structural attenuation in deep reconstruction. As shown in the blue box in Figure 2, the SE adopts a multi-level cascaded convolutional structure composed of three groups of 3 × 3 convolutional layers, each followed by a ReLU activation function. Two spatial attention modules are interleaved in the processing pipeline, as illustrated in Figure 3, to enhance the perception of edges and contours. Specifically, the module applies channel-wise average pooling and max pooling to the input feature map, concatenates the two pooled results along the channel dimension, and then fuses them with a 7 × 7 convolution. A Sigmoid activation is finally used to generate the spatial attention weights $F_{rc}$, which adaptively enhance key structural regions.

**Figure 4:**
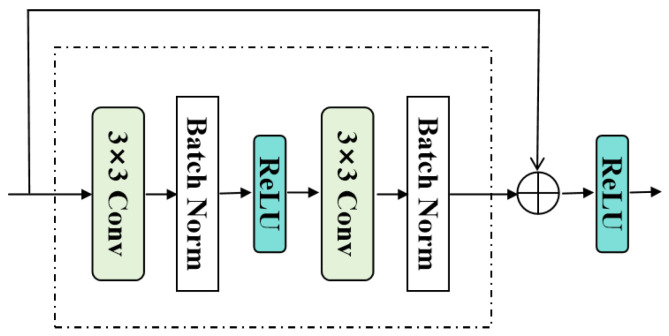
Structure of the residual blocks. Two 3 × 3 convolution layers with batch normalization and ReLU activation are applied in the main path, and a skip connection adds the input to the block output to facilitate stable optimization and feature reuse.

In addition, the SE introduces residual blocks, as shown in Figure 4, to alleviate gradient vanishing and strengthen detail reuse. The main path of each residual block contains two 3 × 3 convolutional layers, each followed by batch normalization, with a ReLU activation inserted between them. A skip connection adds the block input to the main-path output, and the sum is passed through a ReLU activation to obtain the final output, achieving identity mapping to promote gradient flow and feature reuse. The entire structure encoding process can be expressed as follows:

(1) $$F_{\mathrm{arch}}=g_{\mathrm{SA}}\left(f_{3\times 3}\left(\mathrm{ReLU}\left[I_{\mathrm{HR}}\right]\right)\right)$$

### 2.3. Structure Guidance Module (SGM)

As shown in the green box in Figure 2, the structure guidance module (SGM) serves as the key bridge between the structure encoder and the super-resolution network. It delivers structural information to the SGCT backbone through a two-stage injection strategy. In the first stage (feature initialization guidance), the structural features are first rescaled and channel-aligned by interpolation and convolution. They are then fed into a residual sub-block composed of LayerNorm, a 3 × 3 convolution, and ReLU. This suppresses noise and forms a stable shallow structural prior. The output, denoted as $E_{1}$, is injected into the shallow feature $F_{\mathrm{SGCT}1}$ of the super-resolution network. In the second stage (deep multi-level structure guidance), the initialized structural features are sequentially passed through a LayerNorm plus spatial attention module and a LayerNorm plus a multi-scale convolution module (MSConv). The former explicitly enhances responses at edges and contours. The latter integrates structural information at different scales. The resulting features $E_{2}$ and $E_{3}$ are injected into the mid- and deep-level features $F_{\mathrm{SGCT}3}$ and $F_{\mathrm{SGCT}5}$, forming a hierarchical structure feeding process from shallow to deep.

**Figure 5:**
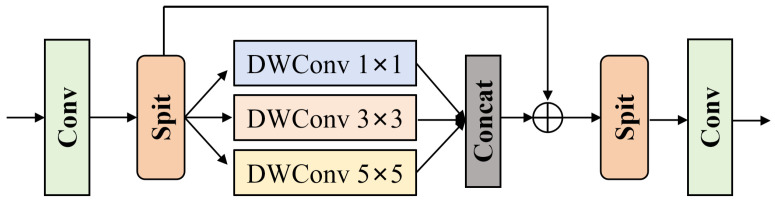
Structure of the MSConv (multi-scale convolution) module. Feature channels are split into parallel multi-scale depthwise branches, capturing structural cues at different receptive fields; the branch outputs are concatenated, fused with a residual connection, and reorganized to reinforce structural information.

The structure of MSConv is shown in Figure 5. The input feature is first processed by a convolution layer to obtain a basic representation. It is then split into three parallel branches along the channel dimension. Each branch uses a depthwise convolution (DWConv) with kernel sizes of 1 × 1, 3 × 3, and 5 × 5 to extract structural details under different receptive fields. The outputs of the three branches are concatenated along the channel dimension and added to the main residual. After that, another channel split and convolution are applied to reorganize the features. In this way, MSConv achieves multi-scale structure modeling while keeping the computational cost under control. The fusion of structural features and backbone features in the SGM can be expressed as follows:

(2) $$F_{\mathrm{fused}}=F_{\mathrm{SGCT}}+MSConv\left(M_{\mathrm{sa}}⊗F_{\mathrm{SE}}^{\mathrm{matched}}\right)$$

$F_{\mathrm{SGCT}}$ denotes the backbone feature at the current layer, $F_{\mathrm{SE}}^{\mathrm{matched}}$ is the structural feature aligned in scale and channels through interpolation and convolution, $M_{\mathrm{sa}}$ is the spatial attention weight map generated by the spatial attention module, MSConv(·) denotes the multi-scale convolutional transform, and ⊗ denotes element-wise multiplication.

This multi-level feature mapping $\left\{E_{1},E_{2},E_{3}\right\}\to \left\{F_{\mathrm{SGCT}1},F_{\mathrm{SGCT}3},F_{\mathrm{SGCT}5}\right\}$ enables precise transmission of structural information. The staged multi-level injection strategy effectively alleviates the information bottleneck of the Transformer backbone in deep structural modeling, ensuring that the skeletal information extracted by the structure encoder can still be fully exploited in the deeper layers of the network.

### 2.4. Super-Resolution Reconstruction Network SGCT

As the backbone of the entire super-resolution reconstruction framework, the SGCT consists of three main modules—shallow feature extraction, deep feature extraction, and image reconstruction—forming an end-to-end pipeline, as shown in the orange part of Figure 2. The shallow feature extraction module uses a 3 × 3 convolution to obtain initial features from the low-resolution input and incorporates feature initialization guidance from the structure guidance module, so that shallow structural priors from the structure encoder are directly injected into this stage.

**Figure 6:**
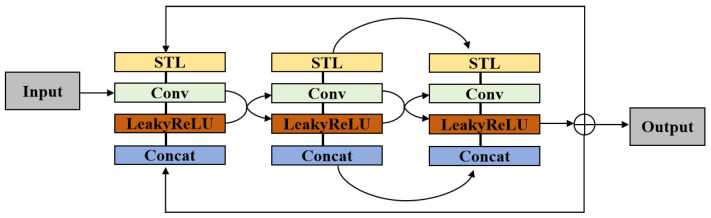
Structure of the proposed DBRDG. Dense feature aggregation is realized by cascaded STL–Conv–LeakyReLU units with progressive feature concatenation, while a group-level skip connection forwards the input to stabilize deep propagation and preserve structural cues.

The deep feature extraction module is built from multiple cascaded dual-branch residual dense groups (DBRDGs), which form the core of the SGCT. As shown in Figure 6, each DBRDG is organized with two cooperative branches. One branch is a dense feature aggregation branch composed of several sequential units, and each unit consists of an STL block followed by a convolution layer and a LeakyReLU activation. The intermediate features from different units are progressively aggregated through dense concatenation to promote feature reuse and strengthen local representation. The other branch is a group-level residual shortcut that directly propagates the input feature and adds it to the aggregated output, which stabilizes optimization and preserves low-frequency information. By stacking multiple DBRDGs, the backbone is able to enhance fine-grained details while maintaining stable feature propagation. After the DBRDG stack, the features are further processed by Transformer-style sub-blocks, including a LayerNorm–W-MSA block and a LayerNorm–MLP block, together with a global residual branch. The window-based multi-head self-attention uses a 16 × 16 window with an overlap ratio of 0.5 to capture long-range dependencies and global structural relationships in remote sensing scenes. Throughout the deep feature extraction stage, multi-level structural features from the structure guidance module are injected into the corresponding layers, so that structural priors can be continuously exploited from shallow to deep features during reconstruction. Different from the dual-branch designs in CRefDiff [39] and DBFNet [40], our two paths are not modality- or domain-specific streams. One path densely aggregates intermediate features to reinforce structural cues, while the other directly forwards the input features through a skip connection to stabilize feature propagation, which helps preserve structure-sensitive patterns in remote sensing imagery.

SRCT is innovative in its tailored design for the unique characteristics of remote sensing images. For the coexistence of regular geometric structures (such as building contours and road networks) and irregular textures (such as vegetation and water bodies), it uses a dual-path structure of window attention and lightweight convolutions to balance global structure modeling and local texture preservation. For the diversity of object types and large scale variations in remote sensing imagery, it designs a multi-scale structural feature injection mechanism to ensure that structures at different scales can be effectively recovered. For the coexistence of large homogeneous regions and rich high-frequency details, it combines a structure guidance module with deep feature fusion to enhance the network’s sensitivity to edges and textures. These targeted designs make SRCT particularly suitable for handling complex object structures and multi-scale features in remote sensing images, enabling it to recover rich texture details while maintaining structural integrity.

To ensure that structural information is effectively preserved throughout the reconstruction process, this study introduces a structural consistency loss:

(3) $$L_{\mathrm{struct}}=\sum_{i=1}^{3}w_{i}\left|\right|E_{i}\left(I_{\mathrm{degraded}}\right)-E_{i}\left(D\left(I_{\mathrm{LR}}\right)\right){\left|\right|}_{1}$$

where $E_{i}$ denotes the *i*-th layer of the structure encoder, *D* denotes bicubic upsampling, and the weighting coefficients $w_{i}$ are set to {0.2,0.6,0.2} to emphasize the importance of mid-level features. This loss term enforces consistency between the structural encodings before and after degradation, providing effective guidance for super-resolution reconstruction.

The complete training and inference workflow of SRCT is illustrated in Algorithm 1. During training, we adopt a feature-space supervision strategy based on the structure encoder. Specifically, both the super-resolved output and the original high-resolution image are passed through a pre-trained structure encoder to extract feature representations, and the L1 loss is then computed in the feature space. Compared with traditional pixel-space losses, feature-space supervision focuses on the semantic content and visually important structures of the image rather than simple pixel-level matching. This supervision enables the model to produce super-resolved results that are more natural in appearance and richer in detail, while avoiding excessive smoothing. The total loss function $L_{\mathrm{total}}$ consists of three parts:

(4) Ltotal=Lrec+λper·Lper+λstr·Lstr

where, $L_{\mathrm{rec}}$ is the L1 reconstruction loss, which ensures accurate pixel-level reconstruction; $L_{\mathrm{per}}$ is the perceptual loss based on the VGG network, which enhances visual perceptual quality; and $L_{\mathrm{str}}$ is the structural feature loss, which effectively preserves structural information through feature-space supervision of the structure encoder.
**Algorithm 1** Training and inference pipeline of SRCT**Input:** HR image set $X_{HR}$, LR image set $X_{LR}$, structure encoder SE,   structure guidance mechanism SGM, backbone SGCT,   loss weights $\lambda_{\mathrm{per}},\lambda_{\mathrm{feat}}$
**Output:** Trained SRCT model; SR prediction for given LR input
1   Initialize SE, SGM and SGCT parameters
2   for epoch = 1 …total_epochs do
3      for batch (xHR, xLR) in (XHR, XLR) do
4         // Phase 1: Structure encoding
5         fSE = SE(xHR)
6         Farch = gSA(fSE)
7         // Phase 2: Structure guidance
8         (E1x, E2x, E3x) = SGM(Farch)
9         Inject E1x, E2x, E3x =⇒ FSRCT1, FSRCT3, FSRCT5
10         // Phase 3: Structure-guided SR reconstruction
11         F0 = Conv3 × 3(xLR)
12         Fdeep = SGRCT(F0, E1x, E2x, E3x)
13         xSR = Upsample(Fdeep)
14         // Loss computation
15         Lrec = ||xSR − xHR||_1_
16         Lper = VGG(xSR, xHR)
17         Lstr = ||SE(xSR) − SE(xHR)||_1_
18         Ltotal = Lrec + λ per · Lper + λ str · Lstr
19         Backpropagate Ltotal, update parameters of SE, SGM, SGCT
20      end for
21   end for
22   // Inference
23   Given unseen LR image xLR*
24   fSE* = SE(HR_ref) or skip if unavailable
25   (E1*, E2*, E3*) = SGM(fSE*) or cached guidance
26   xSR* = SGRCT(xLR*, E1*, E2*, E3*)
27   return xSR*


## 3. Experiment and Results

### 3.1. Dataset

This study uses optical remote sensing images from China’s Gaofen series satellites as experimental data, as shown in Table 1, including the High-Resolution Multi-Mode Imaging Satellite (GFDM), Gaofen-1 (GF-1), and Gaofen-6 (GF-6). The panchromatic images of GFDM have a spatial resolution of 0.5 m and the multispectral images have a resolution of 2 m, while the panchromatic images of GF-1 and GF-6 have a resolution of 2 m and their multispectral images have a resolution of 8 m. The collected scenes cover multiple regions in China, including Beijing, Henan, and Guangxi, and encompass diverse landforms and land-cover types. The imagery was selected from different seasons to capture seasonal appearance variations and typical land-cover changes.

The data preprocessing pipeline comprises four main steps. First, inter-band registration is performed. Second, data fusion is conducted: the 0.5 m panchromatic images and 2 m multispectral images from the GFDM satellite are fused to generate 0.5 m multispectral images as the high-resolution reference, while the 2 m panchromatic images and 8 m multispectral images from GF-1 and GF-6 are fused to obtain 2 m multispectral images as the low-resolution input. Third, orthorectification is applied to the fused images to eliminate geometric distortions caused by terrain relief and satellite viewing angles. Finally, radiometric correction is performed to remove radiation errors introduced by atmospheric and sensor effects. Resampling operations involved in registration, fusion, and orthorectification may introduce small interpolation-related spectral deviations, which are expected to have a minor impact on super-resolution reconstruction.

**Figure 7:**
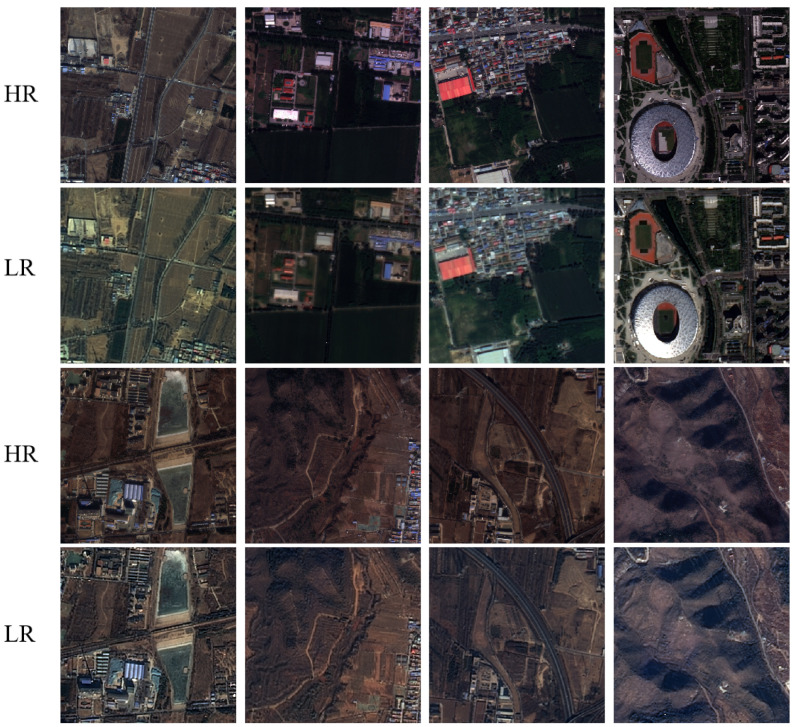
Sample HR–LR image pairs from the dataset.

After the complete preprocessing workflow, the fused data achieve a 4:1 resolution ratio, forming a 4× super-resolution reconstruction dataset. We crop the GFDM imagery into 1600 × 1600-pixel high-resolution patches and the GF-1/GF-6 imagery into corresponding 400 × 400-pixel low-resolution patches, obtaining 8192 paired training samples. The dataset is split into training, validation, and testing sets in a 7:2:1 ratio, and representative samples are shown in Figure 7.

### 3.2. Experimental Details

The experimental settings are summarized in Table 2. For the SRCT model, we trained on 256 × 256 image patches for a total of 300K iterations with a batch size of 4 and an initial learning rate of $1\times 10^{-4}$. We used the Adam optimizer with $\beta_{1}$ = 0.9 and $\beta_{2}$ = 0.99. Data augmentation during training included random rotation and horizontal flipping. All experiments were conducted using PyTorch 1.13.1 on an NVIDIA RTX 3090 GPU with 24 GB of memory.

### 3.3. Evaluation Metrics

To comprehensively evaluate the performance of the proposed method, this study establishes a multi-dimensional evaluation system. The system includes full-reference image quality metrics such as PSNR, SSIM [41], DISTS [42], and FID [43], as well as no-reference image quality metrics such as NIQE [44], MUSIQ [45], MANIQ [46], and CLIPIQA [47]. It covers both traditional evaluation metrics and advanced metrics based on deep learning. The specific formulas and descriptions are provided in Table 3.

### 3.4. Comparison with Other Models

In this study, we selected several advanced super-resolution methods as baselines for comparison. For remote sensing-specific super-resolution, we adopt HAUNet [16], FreMamba [38], and ACT-SR [35]. For general-purpose super-resolution, we choose EDSR [11], CATANet [34], SPSR [24], and WGSR [29] as representative methods. These baselines are selected because they cover diverse modeling paradigms, including CNN-, GAN-, Transformer-, and Mamba-based approaches, and include representative methods that are either classic or recent within each category. Moreover, by including both remote sensing-specific and general-purpose super-resolution models, we provide a more comprehensive comparison.

All compared SOTA methods were implemented under the same software and hardware environment. To ensure fairness, all experiments used the same data augmentation strategy and the default hyperparameter settings for each method. As shown in Table 4, our method achieves the best or near-best performance on multiple key metrics. In terms of perceptual quality, it performs particularly well, with an LPIPS value of 0.2594 and a DISTS value of 0.1625, both being the best among all methods, and a FID value of 77.301, which is also the lowest. This indicates that the distribution of the generated images is closest to that of the real high-resolution images. These results directly validate the effectiveness of the proposed structure-preserving mechanism, demonstrating that our method can retain and transmit critical structural information during degradation and thus address the information loss problem in traditional approaches.

In terms of no-reference quality metrics, our method achieves the best or near-best results on NIQE (5.833), CLIPIQA (0.3776), MUSIQ (42.725), and MANIQA (0.2027), indicating that the generated images have high naturalness and visual quality. This further demonstrates the superiority of our method in real-world scenarios without reference images. Comparing different method types, CNN-based methods such as EDSR and HAUNet tend to produce smooth but detail-deficient results. GAN-based methods such as SPSR and WGSR perform well on some perceptual quality metrics but are less balanced overall than our method. Mamba-based methods such as FreMamba show good balance but do not achieve the best performance on any metric. Transformer-based methods such as ACT-SR and CATANet fall short of our method on structure preservation metrics such as DISTS.

In terms of traditional distortion metrics, our method achieves a PSNR of 24.821 dB and an SSIM of 0.6516, ranking second to some baseline methods. Although it does not achieve the best performance on these traditional metrics, this reflects the common “perception–distortion trade-off” in super-resolution. PSNR and SSIM, which are based on pixel-wise differences and low-level statistics, have inherent limitations for evaluating remote sensing super-resolution. They do not reliably reflect semantic and structural fidelity in complex scenes, as their scores are often dominated by large homogeneous areas and they tend to favor overly smooth reconstructions, making them less sensitive to the structural quality of complex ground objects. To comprehensively demonstrate the performance of each model across multiple types of evaluation metrics, this study normalizes all metrics in the direction where larger values indicate better performance.

For metrics where larger values are better (PSNR, SSIM, CLIP-IQA, MUSIQ, and MANIQA), we use

(5) $$\hat{x}=\frac{x-x_{\min}}{x_{\max}-x_{\min}}$$

For metrics where smaller values are better (LPIPS, DISTS, FID, and NIQE), we use

(6) $$\hat{x}=\frac{x_{\max}-x}{x_{\max}-x_{\min}}$$

**Figure 8:**
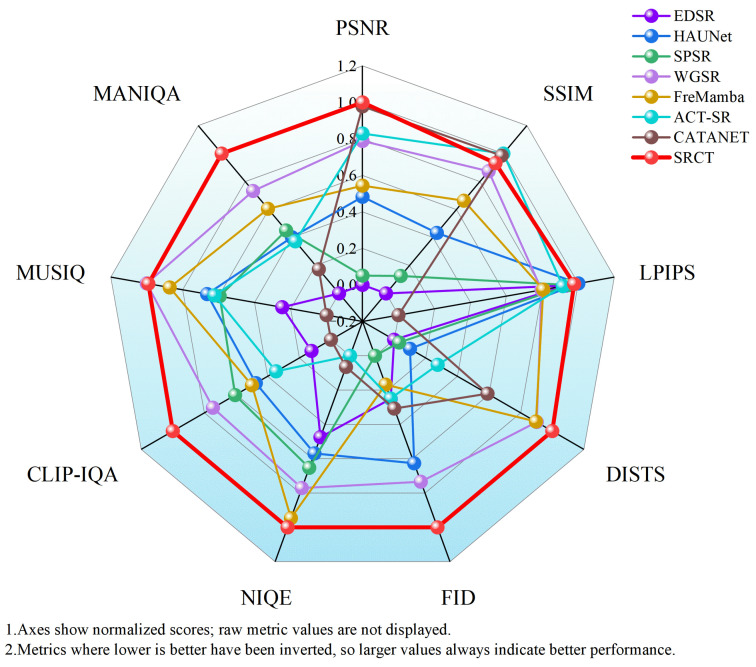
Multi-metric normalized radar chart comparison. The red contour represents the proposed SRCT model. The axes show normalized scores rather than raw metric values. Metrics where lower values indicate better quality are inverted during normalization, so larger values consistently indicate better performance.

The normalized results are shown in Figure 8. SRCT’s contour is closest to the outer ring across nine metrics, indicating superior performance in most evaluation dimensions. Overall, the radar chart shows that SRCT maintains high reconstruction accuracy while balancing structure and perceptual quality, achieving more balanced and comprehensive performance than other methods.

In summary, the experimental results demonstrate the effectiveness and superiority of the proposed structure-preserving super-resolution method for remote sensing image super-resolution, particularly in maintaining the integrity of complex ground object structures.

In addition to quantitative metric comparisons, we conduct visual comparisons to evaluate the reconstruction quality of each method in real remote sensing scenes.

**Figure 9:**
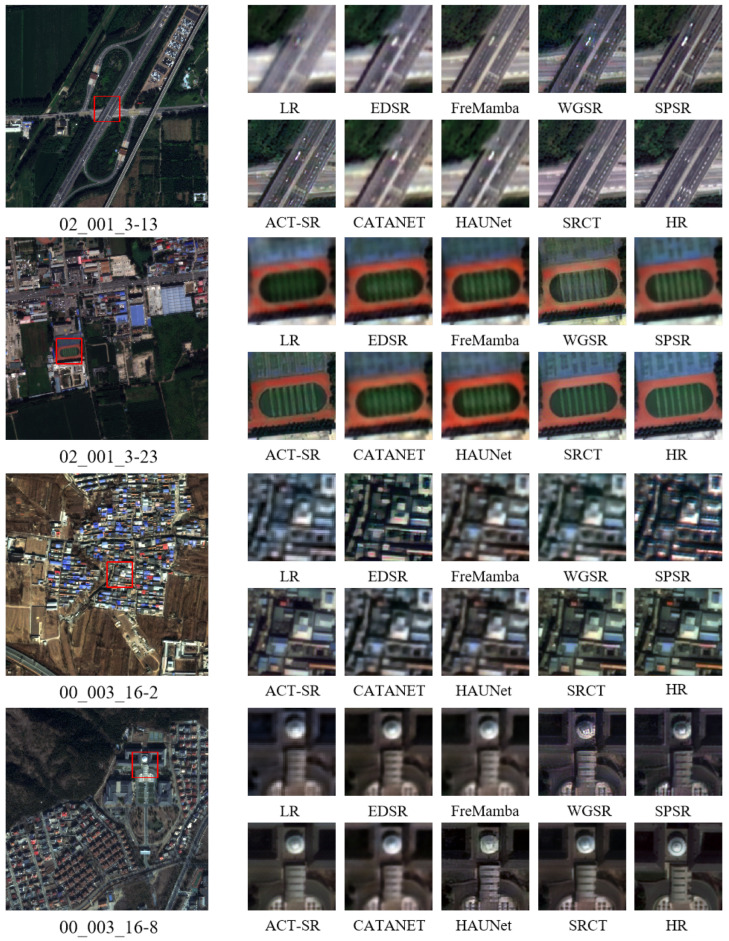
Visual quality comparison results. Visual comparison of SRCT with other models on GFDM (×4). The red box highlights the emphasized region.

As shown in Figure 9, we select typical remote sensing scenes such as roads, playgrounds, and buildings for visual comparison.

In building scenes, EDSR and HAUNet show obvious blurring in building contour recovery, with edges not sharp enough. Although SPSR and WGSR can enhance edge sharpness, they suffer from over-sharpening and false textures, resulting in unnatural texture patterns in roof areas. FreMamba maintains overall structure well but lacks detail processing, with rough roof textures. CATANET and ACT-SR can recover building contours well but exhibit color shifts in roof texture areas, deviating from true colors. In contrast, SRCT accurately reconstructs building geometric contours and roof textures, with clear and natural building edges, and the overall visual effect is closest to real high-resolution images.

In road scenes, EDSR and SPSR show blurring and aliasing artifacts in lane edge recovery, with discontinuous lane lines. Although HAUNet maintains some edge sharpness, it lacks road marking details, with inaccurate marking width and shape. WGSR and FreMamba show discontinuities in lane line recovery, with broken markings. Although CATANET and ACT-SR can sharpen road edges, they introduce random noise, affecting overall visual quality. In contrast, SRCT accurately reconstructs lane markings with continuous and natural lines and smooth road edges with rich details.

In playground scenes, EDSR and CATANET fail to recover track lines clearly, with blurred white line edges. WGSR and SPSR over-sharpen, causing track lines to appear jagged with unnatural edge effects. FreMamba maintains overall structure well but shows broken track markings with incomplete lines. Although ACT-SR can recover the overall playground structure well, detail processing shows distortion with slight geometric shape distortion of the track. In contrast, SRCT completely restores track white lines and lawn stripes, with continuous lines and accurate geometric shapes, maintaining overall structural integrity.

Overall observations show that our proposed SRCT method performs excellently across different types of scenes, maintaining continuous edges, complete structures, realistic colors, and rich details, and significantly outperforms other comparison methods in visual perceptual quality.

### 3.5. Ablation Study

To validate the effectiveness of each component in the proposed framework, we design a series of ablation experiments, as shown in Table 5. Starting from the baseline model, we gradually add three key components—the dual-branch residual dense group (DBRDG), the structure encoder (SE), and the structure guidance module (SGM)—to evaluate the contribution of each component to super-resolution reconstruction performance.

After introducing DBRDG, PSNR increases to 24.2235 dB, SSIM improves to 0.6312, LPIPS decreases to 0.2646, and MANIQA increases to 0.1654, demonstrating the effectiveness of the dual-branch design in balancing global structure modeling and local texture preservation. DBRDG can simultaneously handle regular geometric structures and irregular textures in remote sensing images through the collaborative work of window-based multi-head self-attention in the main path and lightweight convolution in the residual path.

After further introducing the structure encoder (SE), the model performance improves significantly: PSNR reaches 24.8915 dB, SSIM improves to 0.6423, LPIPS slightly increases to 0.2745, DISTS decreases from 0.2123 to 0.1732, and MANIQA increases to 0.1956. This indicates that the structure encoder can effectively extract and preserve key structural information from the original image, providing important structural priors for super-resolution reconstruction. At this stage, structural information is passed to the super-resolution model through feature concatenation, i.e., directly connecting the structural features extracted by SE with the shallow features of the super-resolution network, providing structural priors at the network input stage.

After adding the structure guidance module (SGM), the complete model is formed, and all metrics are further improved. PSNR slightly decreases to 24.821 dB, but SSIM improves to 0.6516, LPIPS decreases to 0.2594, DISTS further decreases to 0.1625, and MANIQA reaches the optimal value of 0.2027. This indicates that SGM successfully injects structural information into all levels of the super-resolution network through the two-stage strategy of feature initialization guidance and deep feature fusion, effectively alleviating the information bottleneck effect in Transformer models. Unlike the single-entry injection when using only the SE, the SGM achieves hierarchical and precise transmission of multi-scale structural features, ensuring that structural information can be effectively utilized at various depth levels of the network. The significant improvement in MANIQA, a metric for evaluating perceptual quality, demonstrates the important role of the SGM in enhancing visual perceptual quality.

The ablation results show that all three proposed core components contribute significantly to model performance. Their synergy enables the method to maintain the integrity of complex ground object structures while recovering rich texture details, making it particularly suitable for handling diverse ground object types and complex scene structures in remote sensing images.

## 4. Discussion

This study focuses on super-resolution reconstruction of sub-meter remote sensing imagery with a spatial resolution of 0.5 m, a resolution range that has been rarely explored in existing research. Although a few works such as SA-GAN [48] and DESAT [49] have attempted to use real remote sensing image pairs for training, their resolutions are still far from the sub-meter level. Sub-meter remote sensing imagery has unique characteristics and challenges. It contains extremely rich ground object details, such as fine building structures, vehicles, and small vegetation, which are often difficult to distinguish in lower-resolution imagery. At the same time, such imagery requires higher edge sharpness and geometric accuracy, making any slight structural distortion more prominent. In addition, the complex coupling between texture and structure is more evident in sub-meter imagery, making it difficult for traditional super-resolution methods to achieve a good balance between detail texture recovery and overall structure preservation.

This study constructs a real image-pair dataset of 0.5 m sub-meter remote sensing imagery and demonstrates the superiority of the proposed method through both quantitative metrics and visual comparisons. In quantitative evaluation, the SRCT model significantly outperforms existing methods on structure-related metrics, with DISTS reduced by 8.7% and LPIPS reduced by 7.2%. Visual comparisons show that SRCT achieves significant improvements in reconstruction quality in structure-sensitive regions such as building contours and road continuity. This study innovatively adopts a strategy combining “external structure guidance” and “internal structure optimization” to comprehensively improve the super-resolution reconstruction quality of sub-meter remote sensing imagery. At the external guidance level, the structure encoder (SE) and structure guidance module (SGM) build a bridge of structural information between high- and low-resolution images. The SE extracts key structural features from high-resolution images through multi-level cascaded convolutions and spatial attention mechanisms, while the SGM precisely delivers these structural features to all levels of the super-resolution network through a two-stage injection strategy. This effectively addresses the problem of structural information degradation in deep networks in traditional methods, enabling complete preservation of complex ground object structures. At the internal optimization level, the dual-branch residual dense group (DBRDG) reshapes the core architecture of the super-resolution network. Through the collaborative work of window-based multi-head self-attention in the main path and lightweight convolution in the residual path, it achieves adaptive processing of remote sensing ground objects. This internal design is particularly suited to the coexistence of regular geometric structures and irregular textures in sub-meter imagery, enabling the model to preserve the integrity of regular structures such as buildings and roads while restoring natural texture details in regions such as vegetation and water bodies. This innovative design combining external and internal mechanisms enables the SRCT model to simultaneously address the dual challenges of structure preservation and texture recovery in sub-meter remote sensing image super-resolution reconstruction, providing a practical and efficient technical solution to the shortage of sub-meter imagery.

**Figure 10:**
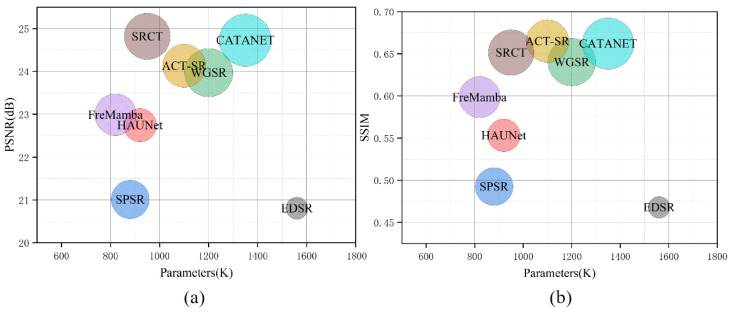
Impact of parameter count and computational complexity on SRCT model performance on the test set. (**a**) Relationship between parameter count and SSIM; (**b**) relationship between parameter count and PSNR, where bubble size represents FLOPs.

This study still has limitations. As shown in Figure 10, SRCT does not have advantages in terms of parameters and FLOPs [50]. Both model size and computational cost per inference are at medium-to-high levels, and the larger bubble also indicates relatively higher FLOPs. Under the same hardware conditions, higher FLOPs typically lead to longer inference time. Nevertheless, SRCT’s actual inference speed and resource consumption remain within acceptable limits. For example, when performing super-resolution reconstruction on a 39,120 × 39,131 GF-6 image of Shouguang City, Shandong Province, China, on an RTX 3090 GPU, SRCT completes the computation in approximately 10 h, corresponding to roughly 23.5 s per megapixel, which can meet the engineering application requirements for sub-meter remote sensing image super-resolution reconstruction. In our operational workflow, the large scene is processed by tiling and mosaicking, including cropping the image into tiles, tile-wise super-resolution inference, adding georeferencing information to the super-resolved tiles, removing overlapping regions, and mosaicking the tiles to generate the final output.

## 5. Conclusions

To address the structure preservation problem in sub-meter remote sensing image super-resolution reconstruction, this paper proposes SRCT, a structure-guided super-resolution method. Through the collaborative work of three core components—the structure encoder (SE), structure guidance module (SGM), and dual-branch residual dense group (DBRDG)—the method achieves the goal of maintaining the integrity of complex ground object structures while recovering texture details. Experimental results show that SRCT significantly outperforms existing methods on structure-related metrics, with DISTS reduced by 8.7% and LPIPS reduced by 7.2%, and achieves notable improvements in reconstruction quality in structure-sensitive regions such as building contours and road continuity. Ablation studies validate the effectiveness of each core component and their synergy. This study provides a new technical approach for sub-meter remote sensing image super-resolution reconstruction and has important application value for addressing the shortage of high-resolution remote sensing data. Future research will further explore the application of structural information in more complex ground object scenarios, optimize model computational efficiency, and deeply integrate the method with specific application domains to promote the application of super-resolution technology in real-world scenarios.

## Figures and Tables

**Table 1:** Spatial resolution of different Gao Fen satellites.

Satellite	PAN Spatial Resolution/m	Multispectral Spatial Resolution/m
GF Multi-Mode	0.5	2
GF-1	2	8
GF-6	2	8

**Table 2:** Experimental settings.

Parameter	Value
Batch size	4
Training patch size	256×256
Iterations	300,000
Learning rate	$1\times 10^{-4}$
Optimizer	Adam, $\beta_{1}=0.9,\;\beta_{2}=0.99$
Data augmentation	Random rotation, horizontal flip
GPU	NVIDIA RTX 3090

**Table 3:** Image quality assessment metrics and descriptions.

Metrics	Formula	Description
PSNR	$\mathrm{PSNR}\left(I_{\mathrm{GT}},I_{\mathrm{SR}}\right)=10\cdot \log_{10}\left(\frac{255^{2}}{\frac{1}{MN}\sum_{i=1}^{M}\sum_{j=1}^{N}{\left(I_{\mathrm{GT}}\left(i,j\right)-I_{\mathrm{SR}}\left(i,j\right)\right)}^{2}}\right)$	A higher PSNR value indicates that the difference in pixel values between the reconstructed image and the original image is smaller.
SSIM	$\mathrm{SSIM}\left(I_{\mathrm{GT}},I_{\mathrm{SR}}\right)=\frac{\left(2\mu_{\mathrm{GT}}\mu_{\mathrm{SR}}+C_{1}\right)\left(2\sigma_{\mathrm{GT}\mathrm{SR}}+C_{2}\right)}{\left(\mu_{\mathrm{GT}}^{2}+\mu_{\mathrm{SR}}^{2}+C_{1}\right)\left(\sigma_{\mathrm{GT}}^{2}+\sigma_{\mathrm{SR}}^{2}+C_{2}\right)}$	A higher SSIM value shows that the two images are more similar in terms of structural information.
LPIPS	$\mathrm{LPIPS}\left(I_{\mathrm{GT}},I_{\mathrm{SR}}\right)=\sum_{l}\frac{1}{H_{l}W_{l}}\sum_{h,w}{∥w_{l}⊙\left(F_{l}{\left(I_{\mathrm{GT}}\right)}_{h,w}-F_{l}{\left(I_{\mathrm{SR}}\right)}_{h,w}\right)∥}_{2}^{2}$	A lower LPIPS value means that the two images are more similar in terms of human-perceived visual quality.
DISTS	$\mathrm{DISTS}\left(I_{\mathrm{GT}},I_{\mathrm{SR}}\right)=\sum_{i}\alpha_{i}\cdot \left(1-S_{i}\right)+\beta_{i}\cdot D_{i}$	A higher DISTS value represents a higher similarity between the images in terms of structure- and texture-related dimensions.
NIQE	$\mathrm{NIQE}\left(I_{\mathrm{SR}}\right)=\sqrt{{\left(\mu_{\mathrm{SR}}-\mu_{\mathrm{model}}\right)}^{T}{\left(\Sigma_{\mathrm{SR}}+\Sigma_{\mathrm{model}}\right)}^{-1}\left(\mu_{\mathrm{SR}}-\mu_{\mathrm{model}}\right)}$	A lower NIQE value indicates that the statistical properties of the image are closer to the statistical laws of typical natural images.
FID	$\mathrm{FID}\left(I_{\mathrm{GT}},I_{\mathrm{SR}}\right)={∥\mu_{\mathrm{GT}}-\mu_{\mathrm{SR}}∥}^{2}+\mathrm{Tr}\left(\Sigma_{\mathrm{GT}}+\Sigma_{\mathrm{SR}}-2{\left(\Sigma_{\mathrm{GT}}\Sigma_{\mathrm{SR}}\right)}^{1/2}\right)$	A lower FID means the features of the generated/processed image are more similar to the real/reference image.
MUSIQ	$\mathrm{MUSIQ}\left(I_{\mathrm{SR}}\right)=f_{\theta }\left(\left[P_{1}\left(I_{\mathrm{SR}}\right),P_{2}\left(I_{\mathrm{SR}}\right),\;\ldots ,P_{n}\left(I_{\mathrm{SR}}\right)\right]\right)$	A higher MUSIQ score shows that the image has fewer visual flaws like artifacts and clearer details.
MANIQA	$\mathrm{MANIQA}\left(I_{\mathrm{SR}}\right)=g_{\phi }\left(A\left(E\left(I_{\mathrm{SR}}\right)\right)\right)$	A higher MANIQA score means the image has a more coherent structure, richer details, and fewer distortions.
CLIPIQA	$\mathrm{CLIPIQA}\left(I_{\mathrm{SR}}\right)={\mathrm{CLIP}}_{\mathrm{high}}\left(I_{\mathrm{SR}}\right)-{\mathrm{CLIP}}_{\mathrm{low}}\left(I_{\mathrm{SR}}\right)$	A higher CLIPIQA score shows that the image has more qualities that match what humans consider a “high-quality image”.

**Table 4:** Performance evaluation results of different models on the test set. Red indicates the best values, blue indicates the second-best values, ↑ indicates higher is better, and ↓ indicates lower is better.

Type	Method	PSNR ↑	SSIM ↑	LPIPS ↓	DISTS ↓	FID ↓	NIQE ↓	CLIPIQA ↑	MUSIQ ↑	MANIQA ↑
CNN-based	EDSR	20.809	0.468	0.2721	0.503	96.402	7.208	0.1857	29.492	0.145
	HAUNet	22.746	0.5534	0.2598	0.469	86.810	6.963	0.2629	36.915	0.1682
GAN-based	SPSR	21.0137	0.4926	0.267	0.4924	102.810	6.743	0.2915	35.665	0.171
	WGSR	23.974	0.6402	0.3097	0.1959	84.097	6.432	0.322	42.891	0.1873
Mamba-based	FreMamba	22.994	0.5987	0.3074	0.198	98.523	5.9749	0.2681	40.618	0.1799
Transformer-based	ACT-SR	24.835	0.6651	0.2768	0.4094	96.471	8.454	0.2349	36.196	0.1665
	CATANET	24.734	0.6622	0.5313	0.3021	94.945	8.285	0.159	25.092	0.155
	Ours	24.821	0.6516	0.2594	0.1625	77.301	5.833	0.3776	42.725	0.2027

**Table 5:** Ablation study results for key modules of SRCT. Red indicates the best values; ↑ indicates higher is better, ↓ indicates lower is better, 🗸 indicates the module is included, and × indicates the module is removed.

Base	DBRDG	SE	SGM	PSNR ↑	SSIM ↑	LPIPS ↓	DISTS ↓	MANIQA ↑
🗸	×	×	×	23.651	0.6143	0.2748	0.206	0.1524
🗸	🗸	×	×	24.2235	0.6312	0.2646	0.2123	0.1654
🗸	🗸	🗸	×	24.8915	0.6423	0.2745	0.1732	0.1956
🗸	🗸	🗸	🗸	24.821	0.6516	0.2594	0.1625	0.2027

## Data Availability

The original contributions presented in this study are included in the article. Further inquiries can be directed to the corresponding authors.
